# Supplementary material for: A place-based assessment of biodiversity intactness in sub-Saharan Africa
Source: Nature. 2025 Dec 3;649(8095):113–21. doi: 10.1038/s41586-025-09781-7 (PMC12727506; doi:10.1038/s41586-025-09781-7)
Supplement: Supplementary file 1 — Methods on mapping strictly protected areas (Supplementary Methods 1); a summary of possible sources of discrepancy between the IUCN Red List and BII assessments for 20 randomly selected ‘outlier’ species (Supplementary Table 1); and descriptions along with representative images of the nine sub-Saharan African land uses for which experts intactness scores in the bii4africa dataset. [file 41586_2025_9781_MOESM1_ESM.pdf]

nature portfolio

https://doi.org/10.1038/s41586-025-09781-7

Supplementary information

A place-based assessment of biodiversity intactness in sub-Saharan Africa

In the format provided by the authors and unedited

Supplementary Information

A place-based assessment of Sub-Saharan Africa's biodiversity intactness

Hayley S. Clements*, Reinette Biggs, Alta De Vos, Emmanuel Do Linh San, Gareth Hempson, Birthe Linden, Bryan Maritz, Ara Monadjem, Chevonne Reynolds, Frances Siebert, Nicola Stevens, Matthew Child, Enrico Di Minin, Karen J. Esler, Maike Hamann, Ty Loft, Belinda Reyers, Odirilwe Selomane, Geethen Singh, Andrew L. Skowno

*Corresponding author: hayleyclements@sun.ac.za

Table of contents

Supplementary Methods 1. Mapping strictly protected areas 1

Supplementary Table 1. Summary of possible sources of discrepancy between the IUCN Red List and Biodiversity Intactness Index (BII) assessments for a random set of ten Critically Endangered species and ten Least Concern species with BII scores in the top and bottom 10th percentile of all vertebrates, respectively. 2

Supplementary Table 2. Descriptions along with representative images of the nine sub-Saharan African land uses for which 200 experts estimated the remaining proportion of an 'intact' reference population, for diverse groups of species in the bii4africa dataset 4

References 6

Supplementary Methods 1. Mapping strictly protected areas

One of the nine land uses for which experts estimated intactness scores was "*Strictly protected areas that generally do not allow for permanent settlements or resource use, though sometimes allow tourism including limited accommodation and road infrastructure. Minimal recent human impact on structure, composition or function of the ecosystem*" (Supplementary Table 2).

We made use of the World Database on Protected Areas[95] to map this land use, limited to protected areas with International Union for the Conservation of Nature (IUCN) management categories I-III. These categories are for more strictly protected areas that limit human infrastructure and resource use[73] thus mirroring the description given to experts.

Four sub-Saharan countries do not use IUCN management categories. For these countries, strictly protected areas were mapped as follows:

Djibouti has two protected areas, both of which are focused on a single species and thus most appropriate to IUCN category IV and therefore excluded.

Guinea-Bissau has 11 protected areas. Eight of these are national parks or Ramsar sites that meet the definition for IUCN category II and are therefore included. The remaining three are migratory corridors that do not meet IUCN category I, II or III definitions and are therefore excluded.

Liberia has three protected areas, all of which are national parks that meet the definition for IUCN category II and are therefore included.

South Africa has several thousand protected areas of different types defined by legislation (e.g., national park, nature reserve, protected environment, etc). Previous studies[96,97] have assigned these types into IUCN management categories based on the regulations and management pertaining to each, and we make use of those allocations to select protected areas under the equivalent of IUCN management categories I-III for inclusion in this study.

Supplementary Table 1. Summary of possible sources of discrepancy between the IUCN Red List and Biodiversity Intactness Index (BII) assessments for a random set of ten Critically Endangered species and ten Least Concern species with BII scores in the top and bottom 10th percentile of all vertebrates, respectively. Coloured blocks indicate species for which the BII or Red List assessment is likely too optimistic (i.e., BII overestimated or threat status underestimated—orange) or pessimistic (i.e., BII underestimated or threat status overestimated—green). Half of the species in this table are poorly known, with unknown population trends or threats. The Critically Endangered species have very localised populations—all but one have just one known location, usually largely or entirely within protected areas (PAs), often where efficacy is questioned or threats are evident in the surrounds. Where efficacy is questioned, our BII assessment likely underestimates threats such as hunting, given the absence of PA effectiveness data across the region. Where the PA is effective, but threats occur in the surrounds, our BII assessment is likely appropriate, noting that a measure of current intactness is not synonymous with a measure of extinction risk. The Least Concern species include five poorly known species, with our BII assessment finding croplands or high-intensity rangelands across a notable portion of each of these species' ranges, suggesting they may be more threatened than currently known. BII is predicted to be low for *A. melampus* and *T. strepsiceros* given the human activities across their very large ranges, while they also are not at risk of extinction since they remain abundant in multiple conservation areas. This is an example of where measures of BII and threat are not synonymous. Another large mammal *C. gnou* is also relatively wide-ranging across grasslands (one of the least intact biomes) where wildlife ranches contribute to a population recovery. Here, our assessment may underestimate BII since most wildlife ranches are *de facto* PAs and thus omitted from our 'strictly protected' land use. *A. aureus* has the majority of its range outside of the BII assessment area, where it may be less exposed to threats than in its sub-Saharan range, resulting in a lower threat status than its regional BII would suggest. Finally, we find croplands around the PA at the core of *S. norae's* localised occurrence, suggesting it may be more threatened than currently known.

Species	BII	Population trend	Range (km^2)	Known locations	IUCN Red List Summary
**Critically Endangered species with high (>90th percentile) BII scores**					
Balebreviceps hillmani - Amphibia Bale Mountains Treefrog	100%	decreasing	5	1	Extremely localised species occurring entirely within protected lands, but with encroachment (livestock, harvesting) into those lands[98].
Arthroleptella subvoce - Amphibia Northern Moss Frog	90%	unknown	30	3	Extremely localised and poorly known species occurring largely within protected lands, which currently have low densities of alien plant species and an acceptable fire-return interval, but these are threats in the area[99].
Piliocolobus pennantii - Mammalia Pennant's Red Colobus	92%	decreasing	73	1	Extremely localised species occurring entirely within protected lands, though the efficacy of that protection is questioned with hunting being a major threat[100].
Hyperolius watsonae - Amphibia	85%	decreasing	5	1	Extremely localised species occurring entirely within protected lands, but with the habitat at risk from harvesting of wood and small-scale agriculture in the surrounds[101].
Ericabatrachus baleensis - Amphibia Bale Mountains Frog	100%	unknown	1	1	Extremely localised and poorly known species occurring entirely within protected lands, but with encroachment (livestock, harvesting) into those lands[102].
Hipposideros lamottei - Mammalia Lamotte's Roundleaf Bat	91%	decreasing	57	1	Extremely localised species with some of its range within protected lands, though its only known roosts are within a mining concession[103].
Myosorex eisentrauti - Mammalia	97%	unknown	31	1	Extremely localised and poorly known species largely occurring within protected lands[104].

Eisentraut's Mouse Shrew					
Arthroleptis troglodytes - Amphibia<br>Cave Squeaker	100%	unknown	16	1	Extremely localised and poorly known species occurring largely within protected lands, though the efficacy of that protection is unknown[105].
Werneria iboundji- Amphibia	87%	decreasing	6	1	Extremely localised species in reportedly intact habitat but with logging threats nearby[106].
Callulina kanga - Amphibia	88%	unknown	25	1	Extremely localised and poorly known species occurring entirely within protected lands in reportedly intact habitat but with growing human populations nearby[107].
**Least Concern species with low (<10th percentile) BII scores**					
Acanthodactylus aureus - Reptilia<br>Golden Fringe-fingered Lizard	28%⁺	stable	5,485	many	Only a small portion of the range is in sub-Saharan Africa, with the majority in North Africa[108].
Aepyceros melampus - Mammalia<br>Impala	47%	stable	3,678,685	many	Extremely large range and abundant in numerous protected areas, as well as on private wildlife ranches and community conservancies[109].
Tragelaphus strepsiceros - Mammalia<br>Greater Kudu	29%	stable	6,431,153	many	Extremely large range and abundant in numerous protected areas, as well as on private wildlife ranches and community conservancies[110].
Surdisorex norae - Mammalia<br>Aberdare Mole Shrew	36%*	stable	3,005	1	Small range with the only known population occurring within protected lands[111.]
Cacosternum aggestum - Amphibia<br>Klipheuwel Dainty Frog	41%⁺	unknown	7,856	2	Recently discovered species, population trend is unknown, with its temporary breeding pools threatened by draining and pollution due to agriculture[112].
Crocidura nigeriae - Mammalia<br>Nigerian Shrew	30%⁺	unknown	224,329	many	Large range, may be affected by habitat loss, poorly known species[113].
Trioceros affinis - Reptilia<br>Beardless Ethiopian Montane Chameleon	44%⁺	stable	316,547	many	Large range, threats are not well-understood, but the species is tolerant of disturbance and can be abundant in modified habitats[114].
Hemidactylus matschiei - Reptilia<br>Togo Leaf-toed Gecko	36%⁺	unknown	65,819	4	Large range, poorly known species; nothing is known about threats but capable of persisting in secondary habitats[115].
Connochaetes gnou - Mammalia<br>Black Wildebeest	20%	increasing	432,483	many	Large range, while grassland habitat loss remains a threat to this endemic species it is abundant in numerous protected areas, as well as on private wildlife ranches[116].
Tarentola senegambiae - Reptilia	48%⁺	unknown	154,912	many	Large range, poorly known species[117].

⁺*Our BII assessment documents croplands and/or high-intensity rangelands across much of the species' range.*
**Our BII assessment documents widespread croplands and high-intensity rangelands surrounding the strictly protected lands within the species' range.*

Supplementary Table 2. Descriptions along with representative images of the nine sub-Saharan African land uses for which 200 experts estimated the remaining proportion of an 'intact' reference population, for diverse groups of species in the bii4africa dataset[5]. Numbers 1 to 6 represent broad land uses, while letters a and b represent respective low and high intensity versions of the associated land use. These land uses were identified by the author team (including expert leads) to capture the major land cover types, uses and associated activities relevant to sub-Saharan Africa (Image source: Unsplash.com)

Land use	Description
1a. Mixed settlements	Suburban areas, smaller towns and rural settlements with large but fragmented human populations interspersed with gardens, parks and near-natural patches of open space, potentially with low densities of cattle, goats, sheep or chickens, or small-scale croplands.
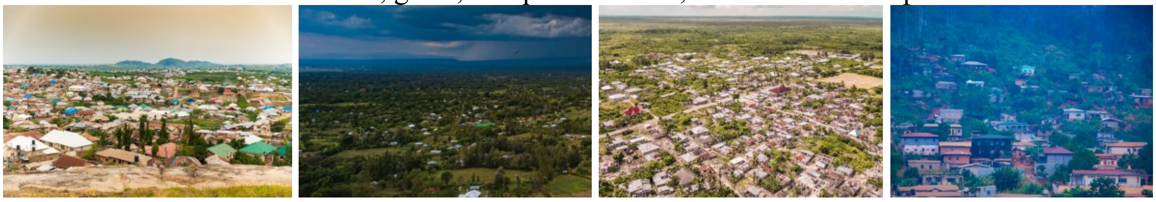	
1b. Dense urban	Densely built-up environments with high human population densities and limited green space — city centres, dense townships, industrial areas, transformed mining areas (e.g., open cast mines, quarries, dumps). Most ecological processes are highly modified. There are few remaining near-natural patches in the landscape, except for e.g., road-side trees and small parks.
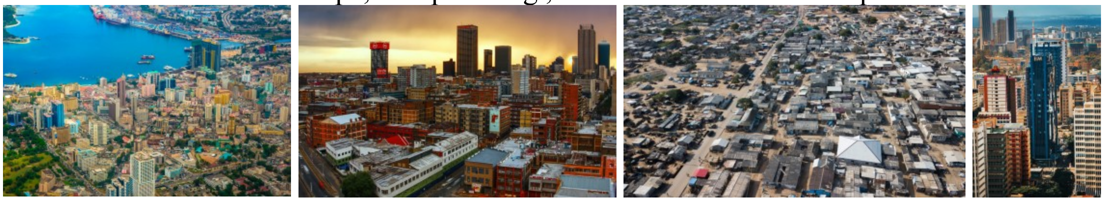	
2. Timber plantations	Lands used for growing trees, typically exotic species, for saw timber, poles or pulp. Harvested by clear-cut every 10 to 30 years, and replanted or regrown from coppice. Includes limited remnant forest, riparian or grassland patches between plantation compartments.
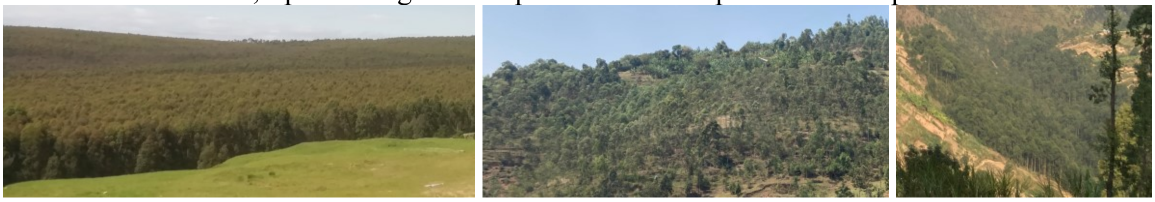	
3a/4a. Non-intensive smallholder croplands	Lands used mainly for smallholder agriculture in small fields (<2 ha), consisting of a diversity of short-duration and long-duration crops (e.g., maize, millet, cassava, beans, squashes, as well as scattered fruit, shade or timber trees). Agricultural inputs of fertilisers and pesticides are very low if any, cultivation is usually manual, there is little or no ploughing or irrigation, and harvest is staggered in time. Fields and homesteads are interspersed with patches of near-natural vegetation. These lands often also support low densities of livestock or smallstock, which are partly free-roaming, and may have semi-natural grazing areas in addition to eating crop residues and cut forage.
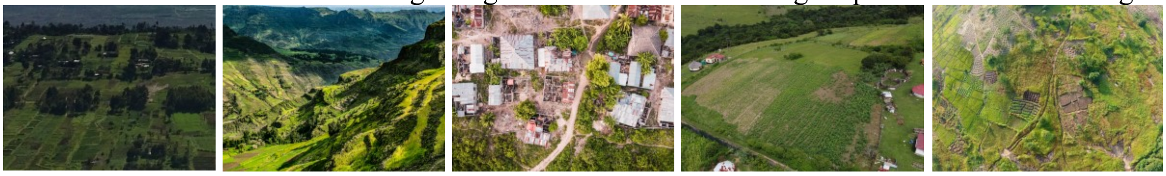	

3b. Tree crop (fruit) plantations

Lands used mainly for tree crops including fruit-bearing tree or shrub plantations (e.g., bananas, coffee, oil palm, cacao, oranges, vineyards, nuts). Non-transformational harvest, usually only the fruit is taken, and trees may be replaced at some stage. Includes limited remnant forest, riparian or grassland patches between plantation compartments.

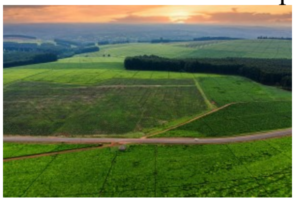
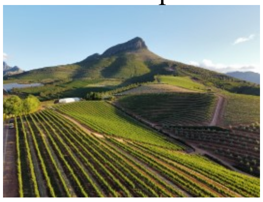
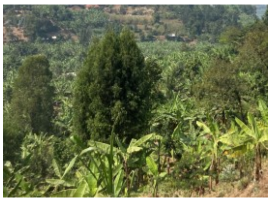
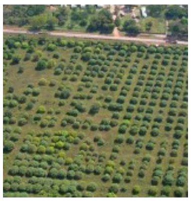

4b. Intensive large-scale croplands

Lands used mainly for short-duration, monocultural crops in large fields (e.g., staple cereal crops, soybeans, sugar cane). Land use activities usually include several of the following: annual ploughing, inorganic fertiliser application, pesticide application, irrigation, mechanisation. When the crop is harvested, the entire biomass is removed and the next crop is planted, perhaps after a fallow period. There are few remaining near-natural patches in the landscape, except for instance on drainage lines, field boundaries and contour strips, or some woodlots or windbreaks of trees.

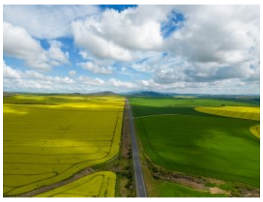
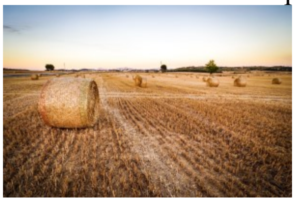
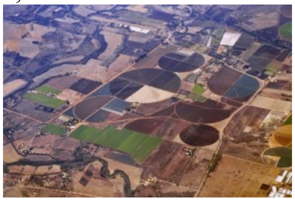

5. Strictly protected areas

Strictly protected areas that generally do not allow for permanent settlements or resource use, though sometimes allow tourism including limited accommodation and road infrastructure (World Database on Protected Area categories I-III or equivalent). Minimal recent human impact on structure, composition and function of the ecosystem.

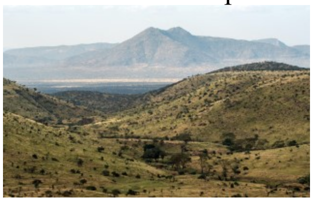
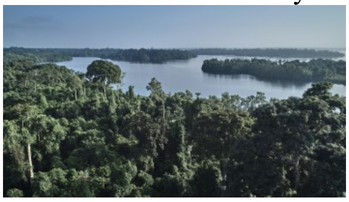
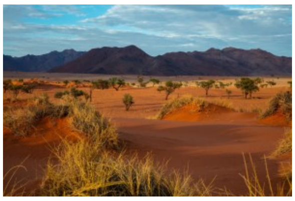

6a. Near-natural lands

Lands (which could be forests, savannas, arid lands, mountainous lands, grasslands) remote from infrastructure, having only minor transformational land use such as crops, planted trees, livestock and human settlements. The human population is relatively low, and livestock or crop-based agriculture or harvest of resources is not at levels that substantially alter natural ecological processes or habitats.

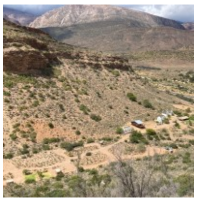
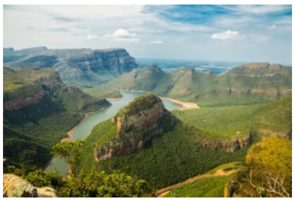
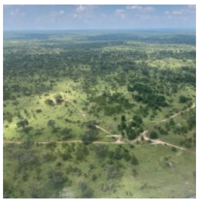
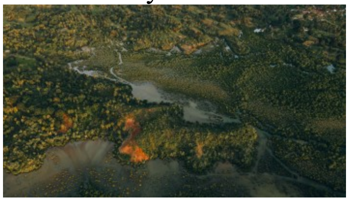

6b. Intensive rangelands

Lands used mainly for livestock grazing either with input of fertiliser or pesticide, or with high stock density relative to what the land can sustain (high enough to cause some disturbance or to stop regeneration of vegetation, or to have done so in the recent past). Domesticated stock such as cattle, sheep, goats are typical, but could also include intensive use of indigenous species such as ostrich.

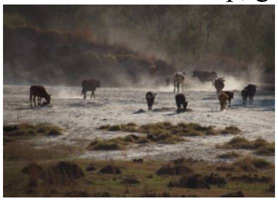
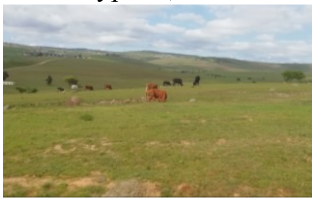
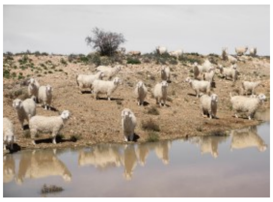
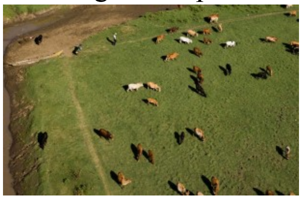

References

96. De Vos, A., Clements, H. S., Biggs, D. & Cumming, G. S. The dynamics of proclaimed privately protected areas in South Africa over 83 years. *Conserv. Lett.* **12**, e12644 (2019).
97. De Vos, A. & Cumming, G. S. The contribution of land tenure diversity to the spatial resilience of protected area networks. *People Nat.* **1**, 331–346 (2019).
98. IUCN SSC Amphibian Specialist Group. 2013. Balebreviceps hillmani. The IUCN Red List of Threatened Species 2013: e.T57703A16948437. http://dx.doi.org/10.2305/IUCN.UK.2013-1.RLTS.T57703A16948437.en
99. IUCN SSC Amphibian Specialist Group & South African Frog Re-assessment Group (SA-FRoG). 2016. Arthroleptella subvoce. The IUCN Red List of Threatened Species 2016: e.T61860A77161647. http://dx.doi.org/10.2305/IUCN.UK.2016-3.RLTS.T61860A77161647.en
100. Cronin, D.T. 2019. Piliocolobus pennantii. The IUCN Red List of Threatened Species 2019: e.T41025A92653653. http://dx.doi.org/10.2305/IUCN.UK.2019-3.RLTS.T41025A92653653.en
101. IUCN SSC Amphibian Specialist Group. 2020. Hyperolius watsonae. The IUCN Red List of Threatened Species 2020: e.T135845A98794815. https://dx.doi.org/10.2305/IUCN.UK.2020-3.RLTS.T135845A98794815.en
102. IUCN SSC Amphibian Specialist Group,. 2013. Ericabatrachus baleensis. The IUCN Red List of Threatened Species 2013: e.T58075A16953634. http://dx.doi.org/10.2305/IUCN.UK.2013-1.RLTS.T58075A16953634.en
103. Monadjem, A. & Cooper-Bohannon, R. 2020. Hipposideros lamottei. The IUCN Red List of Threatened Species 2020: e.T10141A22091938. https://dx.doi.org/10.2305/IUCN.UK.2020-2.RLTS.T10141A22091938.en
104. Gazzard, A., Cronin, D.T. & Hearn, G. 2023. Myosorex eisentrauti. The IUCN Red List of Threatened Species 2023: e.T14106A238804788. https://dx.doi.org/10.2305/IUCN.UK.2023-1.RLTS.T14106A238804788.en
105. IUCN SSC Amphibian Specialist Group. 2016. Arthroleptis troglodytes. The IUCN Red List of Threatened Species 2016: e.T54389A77165733. http://dx.doi.org/10.2305/IUCN.UK.2016-3.RLTS.T54389A77165733.en
106. IUCN SSC Amphibian Specialist Group. 2017. Werneria iboundji. The IUCN Red List of Threatened Species 2017: e.T61761A96225118. http://dx.doi.org/10.2305/IUCN.UK.2017-2.RLTS.T61761A96225118.en
107. IUCN SSC Amphibian Specialist Group,. 2012. Callulina kanga. The IUCN Red List of Threatened Species 2012: e.T193426A2235870. http://dx.doi.org/10.2305/IUCN.UK.2012-1.RLTS.T193426A2235870.en
108. Geniez, P., Mateo, J.A., Joger, U., Pleguezuelos, J., Slimani, T., El Mouden, E.H., Wagner, P. & Wilms, T. 2018. Acanthodactylus aureus. The IUCN Red List of Threatened Species 2018: e.T197434A2483079. http://dx.doi.org/10.2305/IUCN.UK.2018-2.RLTS.T197434A2483079.en
109. IUCN SSC Antelope Specialist Group. 2016. Aepyceros melampus. The IUCN Red List of Threatened Species 2016: e.T550A50180828. http://dx.doi.org/10.2305/IUCN.UK.2016-2.RLTS.T550A50180828.en
110. IUCN SSC Antelope Specialist Group. 2020. Tragelaphus strepsiceros (amended version of 2016 assessment). The IUCN Red List of Threatened Species 2020: e.T22054A166487759. https://dx.doi.org/10.2305/IUCN.UK.2020-1.RLTS.T22054A166487759.en
111. Kennerley, R. 2016. Surdisorex norae. The IUCN Red List of Threatened Species 2016: e.T21134A115160143. http://dx.doi.org/10.2305/IUCN.UK.2016-3.RLTS.T21134A22290472.en
112. IUCN SSC Amphibian Specialist Group, South African Frog Re-assessment Group (SA-FRoG). 2017. Cacosternum aggestum. The IUCN Red List of Threatened Species 2017: e.T76317607A76317868. http://dx.doi.org/10.2305/IUCN.UK.2017-2.RLTS.T76317607A76317868.en
113. Hutterer, R. & Jenkins, P. 2016. Crocidura nigeriae. The IUCN Red List of Threatened Species 2016: e.T41344A115179933. http://dx.doi.org/10.2305/IUCN.UK.2016-3.RLTS.T41344A22307167.en
114. Loader, S. 2014. Trioceros affinis. The IUCN Red List of Threatened Species 2014: e.T203820A2771647. http://dx.doi.org/10.2305/IUCN.UK.2014-3.RLTS.T203820A2771647.en

115. Chirio, L., Trape, J. & Luiselli, L. 2013. Hemidactylus matschiei. The IUCN Red List of Threatened Species 2013: e.T203845A2771815. http://dx.doi.org/10.2305/IUCN.UK.2013-1.RLTS.T203845A2771815.en
116. Vrahimis, S., Grobler, P., Brink, J., Viljoen, P. & Schulze, E. 2017. Connochaetes gnou. The IUCN Red List of Threatened Species 2017: e.T5228A50184962. http://dx.doi.org/10.2305/IUCN.UK.2017- 2.RLTS.T5228A50184962.en
117. Wilms, T. & Wagner, P. 2013. Tarentola senegambiae. The IUCN Red List of Threatened Species 2013: e.T17451414A17451423. http://dx.doi.org/10.2305/IUCN.UK.2013-1.RLTS.T17451414A17451423.en
