# Supplementary material for: A place-based assessment of biodiversity intactness in sub-Saharan Africa
Source: Nature. 2025 Dec 3;649(8095):113–21. doi: 10.1038/s41586-025-09781-7 (PMC12727506; doi:10.1038/s41586-025-09781-7)
Supplement: Supplementary file 2 — Reporting Summary [file 41586_2025_9781_MOESM2_ESM.pdf]

nature portfolio

Corresponding author(s): Hayley Clements

Last updated by author(s): Sep 22, 2025

Reporting Summary

Nature Portfolio wishes to improve the reproducibility of the work that we publish. This form provides structure for consistency and transparency in reporting. For further information on Nature Portfolio policies, see our Editorial Policies and the Editorial Policy Checklist.

Statistics

For all statistical analyses, confirm that the following items are present in the figure legend, table legend, main text, or Methods section.

n/a	Confirmed	
☐	☒	The exact sample size (*n*) for each experimental group/condition, given as a discrete number and unit of measurement
☒	☐	A statement on whether measurements were taken from distinct samples or whether the same sample was measured repeatedly
☐	☒	The statistical test(s) used AND whether they are one- or two-sided<br>*Only common tests should be described solely by name; describe more complex techniques in the Methods section.*
☐	☒	A description of all covariates tested
☐	☒	A description of any assumptions or corrections, such as tests of normality and adjustment for multiple comparisons
☐	☒	A full description of the statistical parameters including central tendency (e.g. means) or other basic estimates (e.g. regression coefficient) AND variation (e.g. standard deviation) or associated estimates of uncertainty (e.g. confidence intervals)
☐	☒	For null hypothesis testing, the test statistic (e.g. *F*, *t*, *r*) with confidence intervals, effect sizes, degrees of freedom and *P* value noted<br>*Give P values as exact values whenever suitable.*
☒	☐	For Bayesian analysis, information on the choice of priors and Markov chain Monte Carlo settings
☒	☐	For hierarchical and complex designs, identification of the appropriate level for tests and full reporting of outcomes
☐	☒	Estimates of effect sizes (e.g. Cohen's *d*, Pearson's *r*), indicating how they were calculated

Our web collection on statistics for biologists contains articles on many of the points above.

Software and code

Policy information about availability of computer code

Data collection	No original data was collected for this study
Data analysis	The land use maps were developed using custom code in Google Earth Engine. The BII maps were developed using custom code in R. All code is available, as outlined in the data availability section.

For manuscripts utilizing custom algorithms or software that are central to the research but not yet described in published literature, software must be made available to editors and reviewers. We strongly encourage code deposition in a community repository (e.g. GitHub). See the Nature Portfolio guidelines for submitting code & software for further information.

Data

Policy information about availability of data

All manuscripts must include a data availability statement. This statement should provide the following information, where applicable:

- Accession codes, unique identifiers, or web links for publicly available datasets
- A description of any restrictions on data availability
- For clinical datasets or third party data, please ensure that the statement adheres to our policy

The expert-elicited bii4africa dataset used in this study is available on Figshare (https://doi.org/10.6084/m9.figshare.c.6710463.v1)5. Input data on species range maps and threat categories are available through the IUCN Red List (https://www.iucnredlist.org/)63 and Birdlife International (http://datazone.birdlife.org/species/requestdis.). Input data on ecoregions are available through Ecoregions2017© Resolve53 (https://ecoregions.appspot.com/). Input data on plant forms in the

RAINBIO65 dataset is available on Github (https://gdauby.github.io/rainbio/). The land use and BII maps generated during this study are available in Figshare (https://figshare.com/s/b3a945793a7c5c4cb9ce), and can be visualised and downloaded on a Google Earth Engine App (https://geethensingh.users.earthengine.app/view/bii).

Research involving human participants, their data, or biological material

Policy information about studies with human participants or human data. See also policy information about sex, gender (identity/presentation), and sexual orientation and race, ethnicity and racism.

Reporting on sex and gender	n/a
Reporting on race, ethnicity, or other socially relevant groupings	n/a
Population characteristics	n/a
Recruitment	n/a Note that this paper makes use of a published dataset co-produced by 200 biodiversity experts, produced as part of the broader project, with recruitment detailed in that publication (Clements, H. S. et al. The bii4africa dataset of faunal and floral population intactness estimates across Africa's major land uses. Scientific Data, https://www.nature.com/articles/s41597-023-02832-6)
Ethics oversight	Ethical clearance for the project was provided by Stellenbosch University (project number 15182).

Note that full information on the approval of the study protocol must also be provided in the manuscript.

Field-specific reporting

Please select the one below that is the best fit for your research. If you are not sure, read the appropriate sections before making your selection.

☐ Life sciences ☐ Behavioural & social sciences ☒ Ecological, evolutionary & environmental sciences

For a reference copy of the document with all sections, see nature.com/documents/nr-reporting-summary-flat.pdf

Ecological, evolutionary & environmental sciences study design

All studies must disclose on these points even when the disclosure is negative.

Study description	We quantified the biodiversity intactness index (BII) across sub-Saharan Africa. We make use of a published bii4africa dataset that contains experts' standardised estimates of the impact of sub-Saharan Africa's predominant land uses on diverse functional groupings of species that represent ~50,000 terrestrial vertebrates and vascular plants. In this paper, we integrate ten spatial datasets to map these land uses, which we combine with bioregional lists of indigenous taxa and the associated bii4africa expert data to map the BII across sub-Saharan Africa.
Research sample	We include all sub-Saharan African terrestrial vertebrate species and plant groups. Species richness per ecoregion was drawn from existing datasets including the IUCN redlist (vertebrates) and Sosef et al 2017 and Kier et al 2005 (plants). Intactness scores for these species were drawn from the published bii4africa dataset (Clements et al 2024).
Sampling strategy	No sampling was done - each pixel across sub-Saharan Africa was included in analyses. Pixel size was selected to match the scale at which experts estimated intactness scores, as described in the paper.
Data collection	No original data were collected. A published dataset, produced as part of the broader project, was used in this study (https://www.nature.com/articles/s41597-023-02832-6) with data collection methods described in detail in that paper, and summarised in the current paper.
Timing and spatial scale	No original data were collected in this study. The published expert dataset that we made us of was produced during expert elicitations that ran from the end of 2020 to the beginning of 2022 (https://www.nature.com/articles/s41597-023-02832-6). The published land use map layers that we used were produced between 1982 (soil map) and 2021 (protected area map), as outlined in Extended Data Table 2. Species range maps were accessed from the IUCN redlist and Birdlife International in 2022 for vertebrates, while plant richness information was drawn from Kier et al 2005 and Sosef et al 2017. Mapping extent was sub-Saharan Africa, including all mainland Afrotropical ecoregions.
Data exclusions	No data were excluded
Reproducibility	n/a - study did not follow an experimental design
Randomization	n/a - no samples were taken
Blinding	Expert data were anonymised during aggregation and analysis, as described in the published dataset that we made use of (https://www.nature.com/articles/s41597-023-02832-6)

Did the study involve field work? ☐ Yes ☒ No

Reporting for specific materials, systems and methods

We require information from authors about some types of materials, experimental systems and methods used in many studies. Here, indicate whether each material, system or method listed is relevant to your study. If you are not sure if a list item applies to your research, read the appropriate section before selecting a response.

Materials & experimental systems

n/a	Involved in the study
☒	☐ Antibodies
☒	☐ Eukaryotic cell lines
☒	☐ Palaeontology and archaeology
☒	☐ Animals and other organisms
☒	☐ Clinical data
☒	☐ Dual use research of concern
☒	☐ Plants

Methods

n/a	Involved in the study
☒	☐ ChIP-seq
☒	☐ Flow cytometry
☒	☐ MRI-based neuroimaging

Plants

Seed stocks	n/a
Novel plant genotypes	n/a
Authentication	n/a
